# Supplementary material for: Efficient Cationization of Cotton for Salt-Free Dyeing by Adjusting Fiber Crystallinity through Alcohol-Water-NaOH Pretreatment
Source: Polymers (Basel). 2022 Dec 18;14(24):5546. doi: 10.3390/polym14245546 (PMC9781206; doi:10.3390/polym14245546)
Supplement: Supplementary file 1 [file polymers-14-05546-s001.zip › polymers-2039877-supplementary.pdf]

Supplementary Information

Efficient Cationization of Cotton for Salt-Free Dyeing by Adjusting fiber Crystallinity through Alcohol-Water-NaOH Pretreatment

Aini Wu, Wei Ma*, Zhiyu Yang, Shufen Zhang

State Key Laboratory of Fine Chemicals, Frontier Science Center for Smart Materials, School of Chemical Engineering, Dalian University of Technology, Dalian 116023, China

Correspondence: weima@dlut.edu.cn; Tel.: +86-411-84986265

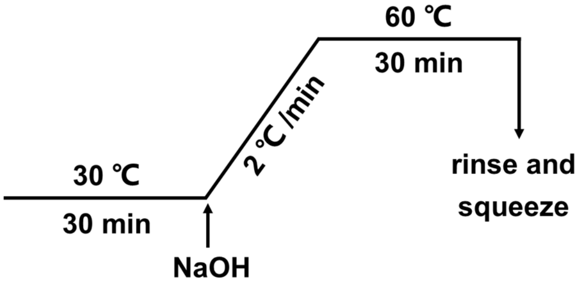

Figure S1. Schematic diagram of the cationization process

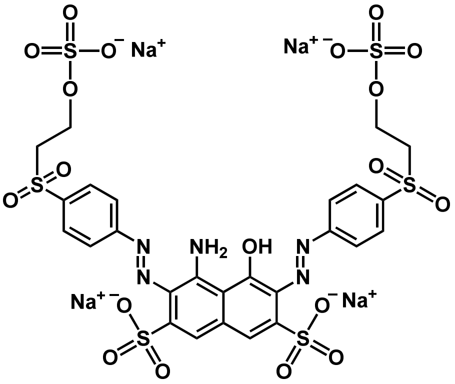

Figure S2. Chemical structure of C. I. Reactive Black 5

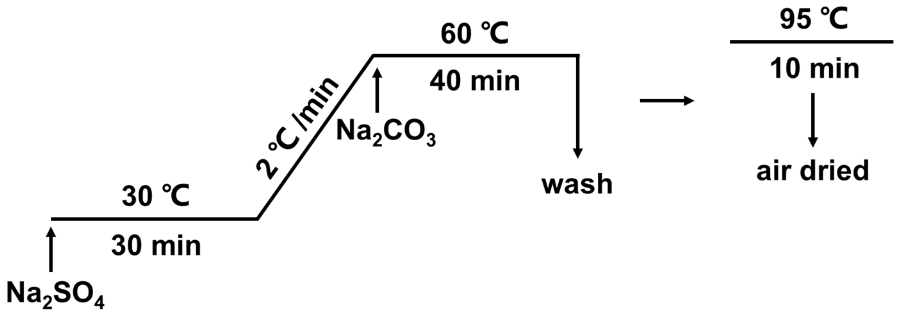

Figure S3. Schematic diagram of the conventional dyeing and soaping process

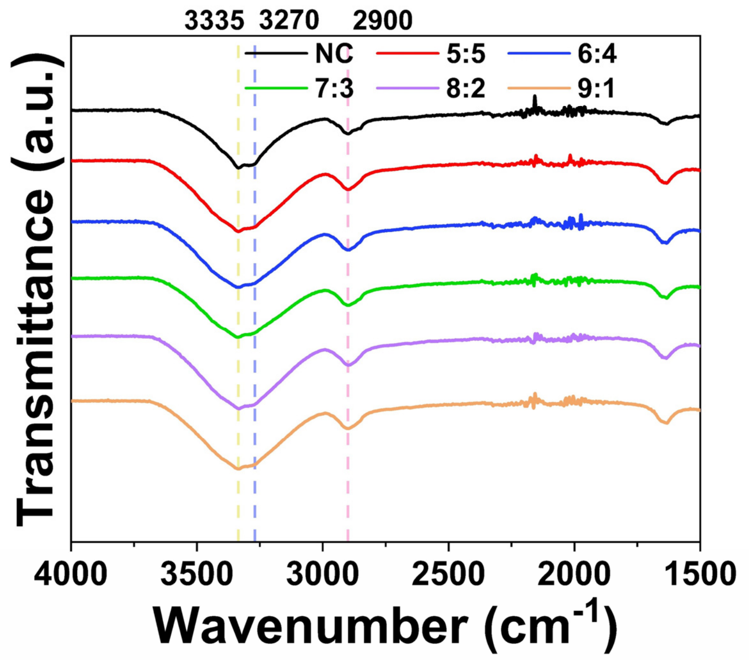

Figure S4. Partial enlargement of Figure 6a

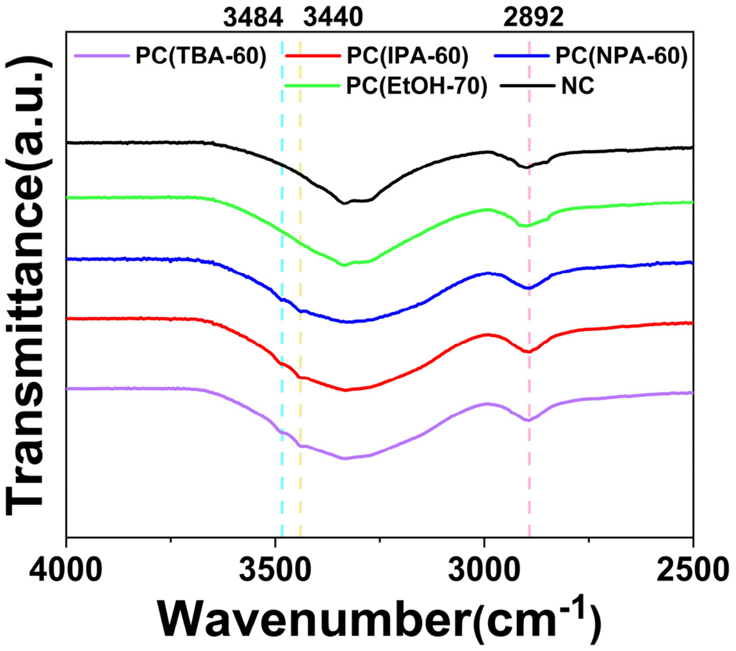

Figure S5. Partial enlargement of Figure 6b

Table S1. Cationic reagent dosage comparison between different references

Cationic agent	Amount (liquid ratio)	Reactive dye usage	Results of salt-free dyeing	References
GTA	60 g/L (1:20)	2% (o.w.f)	F%: 96.74%	Ma, W.; et al. (2017)
CHPTAC	37.5 g/L (Pad-batch)	1.13% (o.w.f.)	E%: >90%	Patiño, A.; et al. (2011)
CHPTAC	80 g/L (1:6)	9.95% (o.w.f)	F%: 58.2%	Arivithamani, N.; et al. (2018)
CHPTAC	30 g/L (1:20)	2% (o.w.f)	E%: 87.68%	Zhang, T.; et al. (2021)
CHPTAC	17.5 g/L (1:17)	1.75% (o.w.f)	E%: 95%	Acharya, S.; et al. (2014)
CHPTAC	40 g/L (1:6)	1% (o.w.f)	E%: 93.64%	Arivithamani, N.; et al. (2016)
GTA	30 g/L (1:3)	6% (o.w.f)	F%: 95.95%	This work

In the listed literatures, when the dye dosage was 1% (o.w.f) to 2% (o.w.f), the required cationic reagent for cationization ranged from 17.5 g/L to 60 g/L, i.e., 0.3 g to 1.2 g of cationic reagent usage per gram of fiber, realizing a dye fixation of higher than

87.68% for salt-free dyeing. Besides, when the dye usage was 9.95% (o.w.f), the cationic reagent addition was 80 g/L, that is, the quantity of the agent consumed was 0.48 g per gram of fiber, achieving a dye fixation of 58.2%.
